# Supplementary material for: Linking deeply-sourced volatile emissions to plateau growth dynamics in southeastern Tibetan Plateau
Source: Nat Commun. 2021 Jul 6;12:4157. doi: 10.1038/s41467-021-24415-y (PMC8260613; doi:10.1038/s41467-021-24415-y)
Supplement: Supplementary file 1 — Supplementary Information [file 41467_2021_24415_MOESM1_ESM.pdf]

Supplementary Information for

Linking deeply-sourced volatile emissions to plateau growth dynamics in southeastern Tibetan Plateau

M. Zhang et al.

This PDF file includes:

Supplementary Methods
Supplementary Fig. 1. Map showing tectonic framework and sampling sites.
Supplementary Fig. 2. Comparison between $^3He/^4He$ distribution and seismic images.
Supplementary Fig. 3. Comparison between $^3He/^4He$ distribution and crustal Vp/Vs data.
Supplementary Fig. 4. Comparison between $^3He/^4He$ distribution and crustal thickness.
Supplementary Fig. 5. Example of multi-component mixing based on He-CO_2 data.
Supplementary Fig. 6. Plot showing elemental fractionation between He and CO_2.
Supplementary References (1–19)

Supplementary Methods

1. Field campaigns and sample collection

The 2011 field campaign focused on the Tengchong volcanic field and a few active faults in Yunnan Province, which is located in southern part of the SETP. Temperature, pH, electrical conductivity (EC), were measured on the site with handheld meters calibrated prior to sampling. Low-He diffusivity glass containers (for free gas sample) and copper tubes (for water sample) were used to collect 24 samples from the Tengchong volcanic field and 6 samples from active faults in eastern Yunnan [see Hilton et al.[1] for sampling details]. In July and August of 2018, we conducted a field campaign to collect fluid samples from active faults in northern part of the SETP with the aim of investigating the volatile emissions in non-volcanic region. Temperature, pH, electrical conductivity (EC), were measured on the site with handheld meters calibrated prior to sampling. Glass bottles (volume = 100 ml) were used to collect 44 samples of gas and water (36 from the Xianshuihe fault and Anninghe fault, and 8 from the Litang fault) following water displacement method. Specifically, a glass bottle fully filled with spring water was placed directly on the bubbles through an inverted funnel submerged in the water, allowing accumulation of bubbles in the bottle to displace the water out. When water was close to be fully displaced, a silicone stopper was used to seal the glass bottle in spring water; and the sampled bottle was kept inverted with a bit of spring water in it to avoid air contamination during transportation to laboratory.

Table showing major information of the investigated drilled well in the Sichuan basin

Sample site	Latitude (N)	Longitude (E)	Elevation (m)	Well depth (m)	Country rocks	Tectonic affiliation	T (°C) /pH	Sample ID	Sample type	Sample container
Daxing	30°14′56.3″	103°24′39.0″	560	1688	Quartz sandstone	Pujiang-Xinjin fault	17.8/nd	DX2001	Water	Glass bottle
Xiangxiu	31°44′04.8″	104°34′32.7″	502	500	Sandstone	Beichuan-Yingxiu fault	14.6/9.8	XX2001-Cu	Water	Copper tube
Beichuan	31°37′08.5″	104°28′16.9″	534	300	Quartz sandstone, Siliceous sandstone	Beichuan-Yingxiu fault	15.6/8.0	BC2001	Water	Glass bottle
Nanxi	28°59′03.0″	104°56′18.5″	286	101	Quartz sandstone	Qingshan anticline	16.1/7.7	NX2001	Water	Glass bottle
Wanfo	31°16′05.5″	104°28′01.6″	553	3072	Quartz sandstone	Baimaguan anticline	16.5/8.4	WF2001	Water	Glass bottle
Luohanchang	28°53′38.3″	105°29′49.6″	209	300	Sandstone, Limestone	Zhongxing anticline	18.0/7.5	LHC2001	Water	Glass bottle
Luohanchang	28°53′38.3″	105°29′49.6″	209	300	Sandstone, Limestone	Zhongxing anticline	18.0/7.5	LHC2001-Cu	Water	Copper tube

Seventeen samples of gas and water were collected during the field campaigns in 2019 and 2020. Temperature, pH, electrical conductivity (EC), were measured on the site with handheld meters calibrated prior to sampling. Lead glass containers, copper tubes and a glass syringe[2] were used to collect gas and water samples from natural springs and drilled wells in the Sichuan basin (details on the drilled wells[3] are given in the above Table). For springs without free gas phase, the lead glass container was fully filled with spring water; and otherwise, the copper tube (length = 20–30 cm) was used to collect water. Two clamps were placed on both ends of the copper tube to minimize fluid interaction with atmosphere. For drilled wells, a rubber tube was submerged into the well to approximately ~10 m below water level of the well, and then water was pulled through the sampling apparatus (lead glass container or copper tube) using a glass syringe for

several times to flush out the air in the silicone rubber tubes before collection of water sample.

2. He isotope correction and calculation

Assuming that all ^{20}Ne of the measured samples originates from air dissolved in water, the air-derived helium contributions were corrected following the equations below:

$$X = (^{4}He/^{20}Ne)_{M}/(^{4}He/^{20}Ne)_{air} \times \beta_{Ne}/\beta_{He} \quad (1)$$

where $(^{4}He/^{20}Ne)_{M}$ is the measured $^{4}He/^{20}Ne$ ratio of the sample, and β represents the Bunsen solubility coefficient assuming a recharge temperature of 15°C[4].

$$R_{C} = [(R_{M}/R_{A} \times X) - 1]/(X - 1) \quad (2)$$

where R_C is air-corrected $^{3}He/^{4}He$, R_M is measured $^{3}He/^{4}He$, and R_A = air $^{3}He/^{4}He = 1.39 \times 10^{-6}$.

Using the air-corrected $^{3}He/^{4}He$ values (reported in R_C/R_A), we calculated the proportions of the mantle He in the total He inventory, assuming a binary mixing model between the depleted mantle ($^{3}He/^{4}He = 8 \pm 1$ R_A[5]) and crust ($^{3}He/^{4}He = 0.02$ R_A[6]):

$$P_{M} = (R_{C} - 0.02)/(8 - 0.02) \quad (3)$$

where P_M represents the proportion of the mantle He, and R_C is air-corrected $^{3}He/^{4}He$.

3. Constraining CO_2 and N_2 inventory using He-C-N data

Given that noble gas isotopes (e.g., $^{3}He/^{4}He$) are effective tracers for the origin of major volatile species such CO_2 and N_2, we present ternary mixing calculations to constrain the proportions of different end-member components involved in the origins of CO_2 and N_2 based on He-C-N elemental/isotopic data.

For CO_2 inventory calculation, the equations[7] are:

$$(^{13}C/^{12}C)_{Obs} = f_{M} \times (^{13}C/^{12}C)_{M} + f_{L} \times (^{13}C/^{12}C)_{L} + f_{S} \times (^{13}C/^{12}C)_{S} \quad (4)$$

$$1/(^{12}C/^{3}He)_{Obs} = f_{M}/(^{12}C/^{3}He)_{M} + f_{L}/(^{12}C/^{3}He)_{L} + f_{S}/(^{12}C/^{3}He)_{S} \quad (5)$$

$$f_{M} + f_{L} + f_{S} = 1 \quad (6)$$

where Obs, M, L, and S represents observed value of samples, MORB, CAR and ORG end-members.

For N_2 inventory calculation, the equations[8] are:

$$(^{15}N/^{14}N)_{Obs} = f_M \times (^{15}N/^{14}N)_M + f_A \times (^{15}N/^{14}N)_A + f_S \times (^{15}N/^{14}N)_S \quad (7)$$

$$1/(N_2/He)_{Obs} = f_M/(N_2/He)_M + f_A/(N_2/He)_A + f_S/(N_2/He)_S \quad (8)$$

$$f_M + f_A + f_S = 1 \quad (9)$$

where Obs, M, A, and S represents observed value of samples, MORB, AIR and ORG end-members.

For the above two sets of equations, reference values[7–9] for parameters of depleted mantle (MORB), carbonate (CAR), organic matter (ORG), and sediments (SED) end-members are as follows:

MORB: $\delta^{13}C = -6.5 \pm 2.5‰$, $\delta^{15}N = -5 \pm 2‰$, $CO_2/^3He = 2 \times 10^9$, $N_2/He = 1.5 \times 10^2$;

CAR: $\delta^{13}C = 0 \pm 2$ ‰, $CO_2/^3He = 10^{13}$;

ORG: $\delta^{13}C = -30 \pm 10‰$, $CO_2/^3He = 10^{13}$;

SED: $\delta^{15}N = 7 \pm 4‰$, $N_2/He = 1.05 \times 10^4$;

AIR: $\delta^{15}N = 0‰$, $N_2/He = 1.489 \times 10^5$.

4. Secondary processes involved in He-CO_2 systematics

The multi-component mixing processes are likely to result in variations in $\delta^{13}C$-CO_2, $CO_2/^3He$, and $^3He/^4He$ values. However, as shown in Supplementary Fig. 5A, many samples have $CO_2/^3He$ ratios lower than that of MORB ($CO_2/^3He = 2 \times 10^9$) and are plotted outside the mixing envelope, suggesting that they cannot be explained by multi-component mixing alone. Therefore, many previous studies have discussed how secondary processes (e.g., calcite precipitation) affect the He-CO_2 isotopic compositions and abundance ratios.

Volatile systematics (e.g., $CO_2/^3He$) of a hydrothermal degassing system are affected by the balance between volatile input from deep source and volatile output to the surface. In a case of continuous hydrothermal degassing without sufficient recharge from the deep (i.e., volatile output > volatile input), the lower solubility of He in aqueous fluids relative to that of CO_2 would lead to low He contents and elevated $CO_2/^3He$ of the residual fluid phase[10,11]. Accordingly, hydrothermal degassing can also cause carbon isotopic fractionation as $^{13}CO_2$ preferentially retains in the residual fluid phase at temperatures <110 °C[12,13], giving rise to isotopically heavier $\delta^{13}C$ values of the residual fluids. As a result, variable degrees of hydrothermal degassing would lead to deviations of $\delta^{13}C$-CO_2,

$CO_2/^3He$, and He content of the sampled gases from corresponding original source values.

Based on the above criteria, the negative correlation between $CO_2/^3He$ and He appears to reflect variable degrees of hydrothermal degassing (Supplementary Fig. 6). In addition, the loss of CO_2 induced by calcite precipitation from hydrothermal fluids is also likely to fractionate both $CO_2/^3He$ and $\delta^{13}C$ values. This is shown by the calcite precipitation line at 25°C and 192 °C in Supplementary Fig. 5A, assuming a starting point of $\delta^{13}C\text{-}CO_2 = -5.4‰$ and $CO_2/^3He = 2.75 \times 10^{10}$. The trend between $CO_2/^3He$ and $\delta^{13}C\text{-}CO_2$ values in Fig. 3A of the main text are likely the results of calcite precipitation. Overall, these secondary processes appear to be common for the samples, and are mainly controlled by the solubility differences in aqueous fluids between CO_2 and He.

5. Calculation of average strain rate of the studied active faults

After compiling strain rate data from Global Strain Rate Model[14], we calculated average strain rates of the studied active faults were calculated based on distribution of the fault system.

For the NS-trending sections of the Three Rivers faults (TRF), the strain rate data within the region of 25°48’−28°00’N, 98°36’−100°12’E were considered. For the NW-SE-trending segments of the TRF, which expand to northwest by 0.1° ×0.1° increment assuming an identical width with the NS-trending segments, the strain rate data within the region of 28°00’−29°18’N, 97°18’−98°54’E. This yields an average strain rate of 17 ± 21 nstrain/yr (1σ, n = 595) for the TRF. For the Litang fault (LTF), the strain rate data within 0.2° longitude to the east and to the west, respectively, from the fault axis were considered, which yield an average strain rate of 30 ± 12 nstrain/yr (1σ, n = 85) for the LTF. For the NS-trending sections of the Xianshuihe fault (XSHF), the strain rate data within the region of 29°18’−30°00’N, 101°30’−102°12’E were considered. For the NW-SE-trending segments of the XSHF, located between 30°00’N and 31°42’N, the strain rate data within 0.2° longitude to the east and to the west, respectively, from the fault axis were considered. This yields an average strain rate of 123 ± 60 nstrain/yr (1σ, n = 149) for the XSHF.

For the Anninghe-Xiaojiang fault (AXF), the strain rate data within 0.2° longitude to the east and to the west, respectively, from the fault axis were considered for the Anninghe fault; and the strain rate data within the region of 23°12’−26°54’N, 102°30’−103°36’E were considered for the Xiaojiang fault. This yields an average strain rate of 58 ± 29 nstrain/yr (1σ, n = 576) for the AXF. For the Bookshelf faults and Red River fault (BF & RRF), the strain rate data within the region of 22°00’−24°12’N, 96°30’−105°00’E and 22°24’−23°18’N, 102°42’−103°54’E were considered, which yield an average strain rate of 30 ± 15 nstrain/yr (1σ, n = 1398) for the BF & RRF.

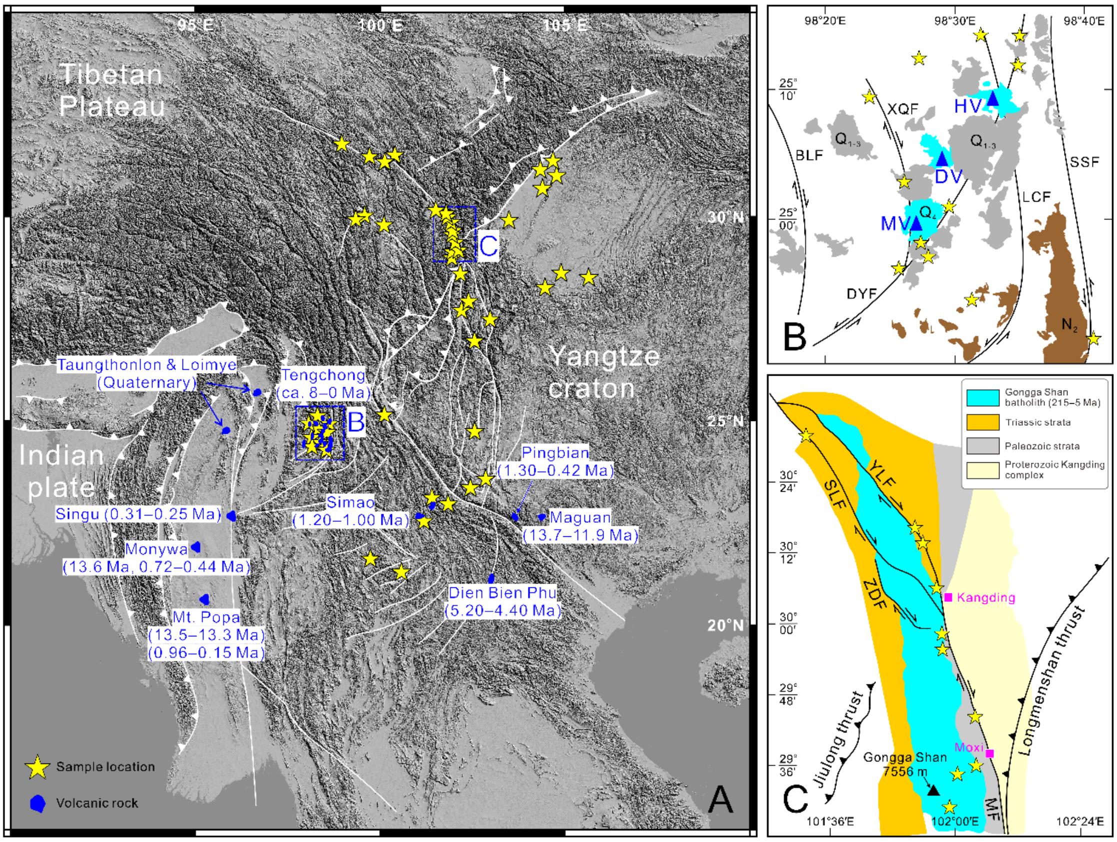

Supplementary Fig. 1. Simplified geological map showing tectonic framework of the SETP and adjacent region and the sample locations in this study. (A) Distribution of active faults, late Cenozoic volcanic rocks, and sample locations in the study area. Ages of the late Cenozoic volcanic rocks are summarized in Supplementary Data 5. (B) Thermal springs, active volcanoes, and late Cenozoic volcanic rocks (N_2, $Q_{1\text{-}3}$ and Q_4) in Tengchong. MV, Maanshan volcano; DV, Dayingshan volcano; HV, Heikongshan volcano; BLF, Binlangjiang fault; XQF, Xinqi fault; DYF, Dayingjiang fault; LCF, Longchuan fault; SSF, Songshan fault. (C) Simplified map of regional geology and sample locations in Kangding-moxi section of the Xianshuihe fault. YLF, Yalahe fault; SLF, Selaha fault; ZDF, Zheduotang fault; MF, Moxi fault. (The map in A is generated by Maoliang Zhang using Global Mapper software).

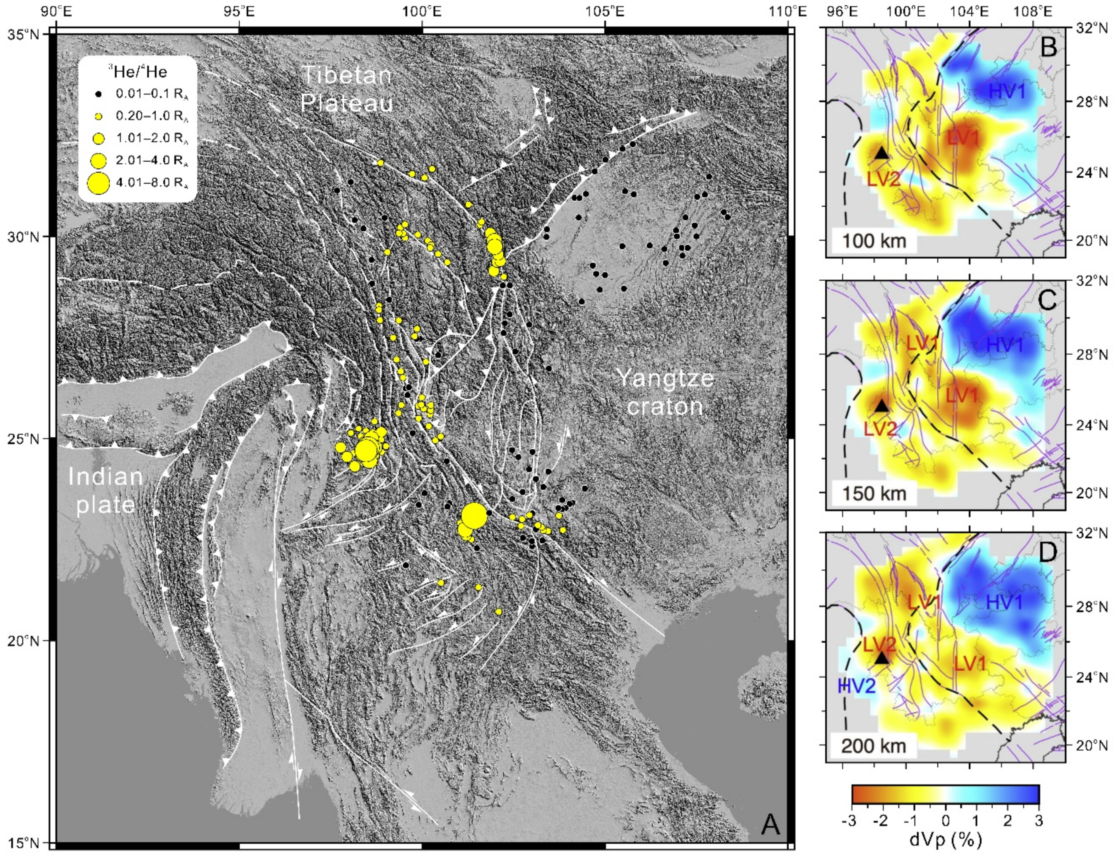

Supplementary Fig. 2. Comparison between ^{3}He/^{4}He distribution and seismic velocity structure. Tectonic framework is given in the main text and caption of Fig. 1. High-resolution P-wave seismic images have revealed low velocity zones at variable mantle depths (65−250 km[15]) beneath the SETP, which are interpreted as possible asthenospheric mantle flows surrounding the Yangtze craton. However, there is no correlation between these high ^{3}He/^{4}He anomalies (A) and low velocity zones (B–D). For example, the crust-dominated ^{3}He/^{4}He values of fluids from the Xiaojiang faults correspond with a clear low velocity zone at 150 km depth (approximately the depth of uppermost asthenospheric mantle beneath the SETP), whereas the Kangding-Moxi region of the Xianshuihe fault is characterized by high ^{3}He/^{4}He values above a lower magnitude of low velocity zone at the 150 km depth. This strongly suggests that the asthenospheric mantle flow cannot account for the ^{3}He/^{4}He distribution in the SETP. (The map in A is generated by Maoliang Zhang using Global Mapper and ArcGIS software).

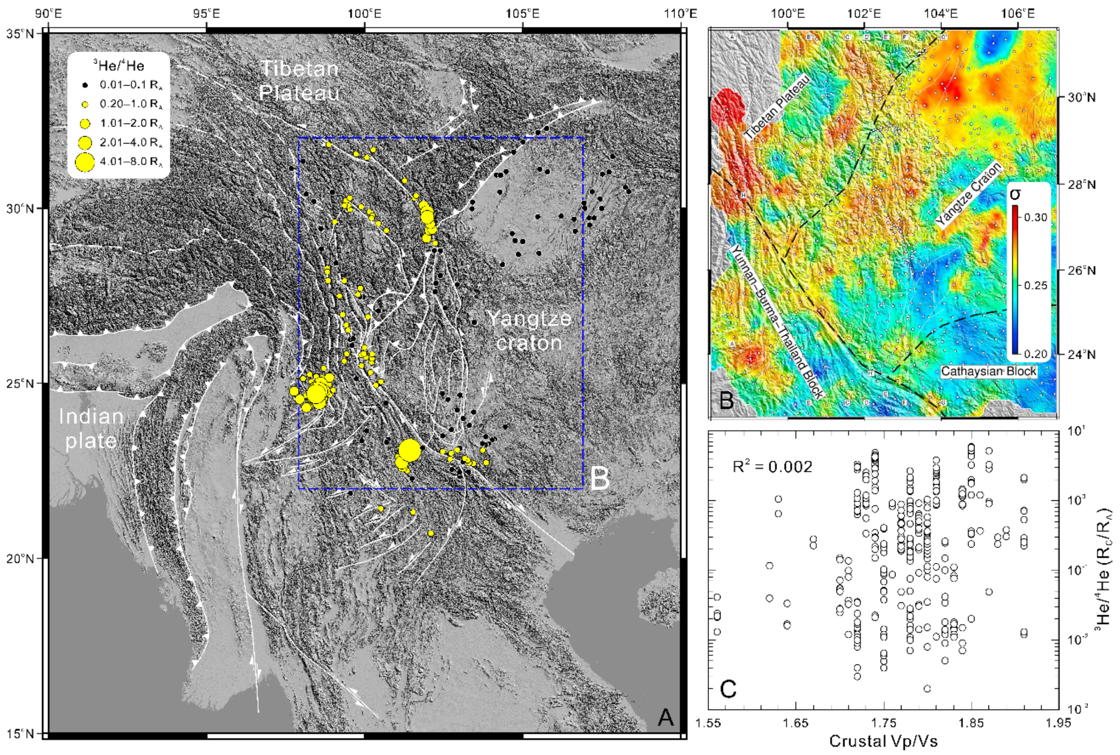

Supplementary Fig. 3. Comparison between ^{3}He/^{4}He distribution and crustal Vp/Vs data. Tectonic framework is given in the main text and caption of Fig. 1. Relationship between Poisson's ratio σ and crustal Vp/Vs is $\sigma = [0.5 \times (Vp/Vs)^2 - 1]/[(Vp/Vs)^2 - 1]$, when σ = 0.20, 0.25 and 0.30, the Vp/Vs would be 1.63, 1.73 and 1.87, respectively. Spatially, there is no correlation between ^{3}He/^{4}He distribution (A) and distribution of the Poisson's ratios[16] (B), as also shown by the very low R^2 value (0.002; C) obtained from regression analysis of the ^{3}He/^{4}He and crustal Vp/Vs data[16,17]. This indicates that crustal lithology does not control ^{3}He/^{4}He distribution in the SETP. (The map in A is generated by Maoliang Zhang using Global Mapper and ArcGIS software).

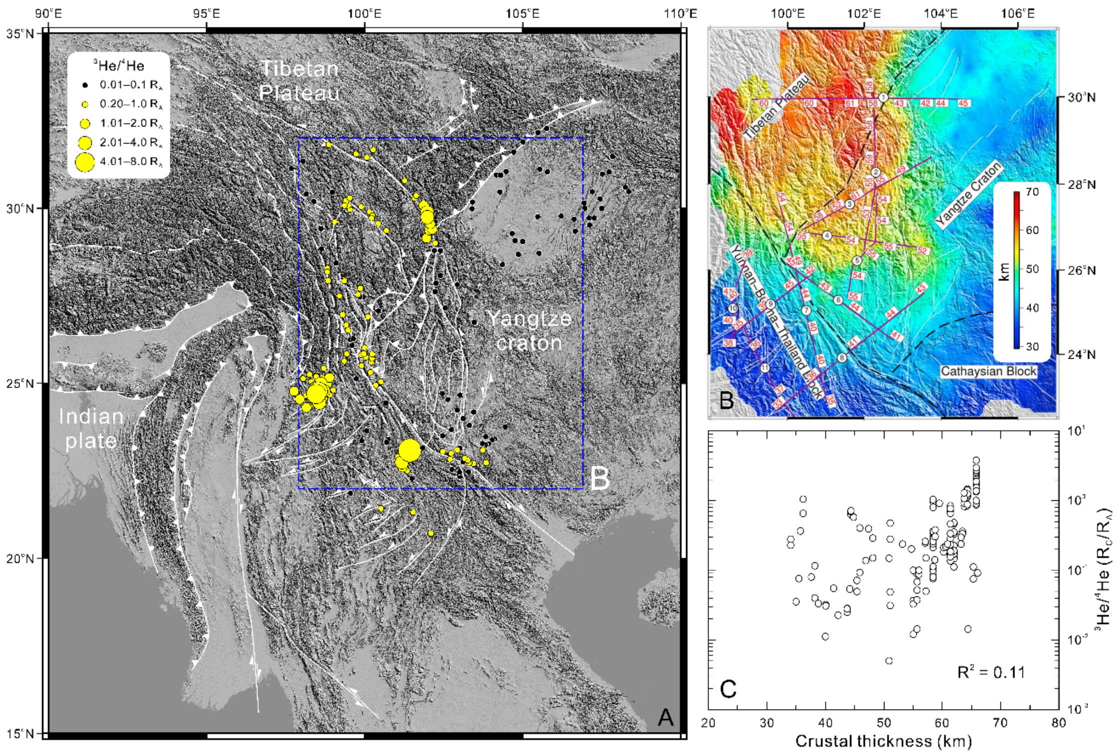

Supplementary Fig. 4. Comparison between ^{3}He/^{4}He distribution and crustal thickness. Tectonic framework is given in the main text and caption of Fig. 1. No spatial correlation between ^{3}He/^{4}He distribution (A) and crustal thickness (B) can be observed[17], which is consistent with low very R^2 value (0.11) obtained from regression analysis of ^{3}He/^{4}He and crustal thickness (C). This would rule out the possibility of crustal thickness as a rough indicator for mantle ^{3}He dilution by radiogenic helium in the crust. The lithospheric thickness data are only available for a small number of sampling sites, but we do not expect statistically meaningful correlations between ^{3}He/^{4}He values and lithospheric thickness for the entire SETP. This is because basically the ^{3}He/^{4}He varies more dramatically from place to place than the corresponding spatial variations in lithospheric thickness. (The map in A is generated by Maoliang Zhang using Global Mapper and ArcGIS software).

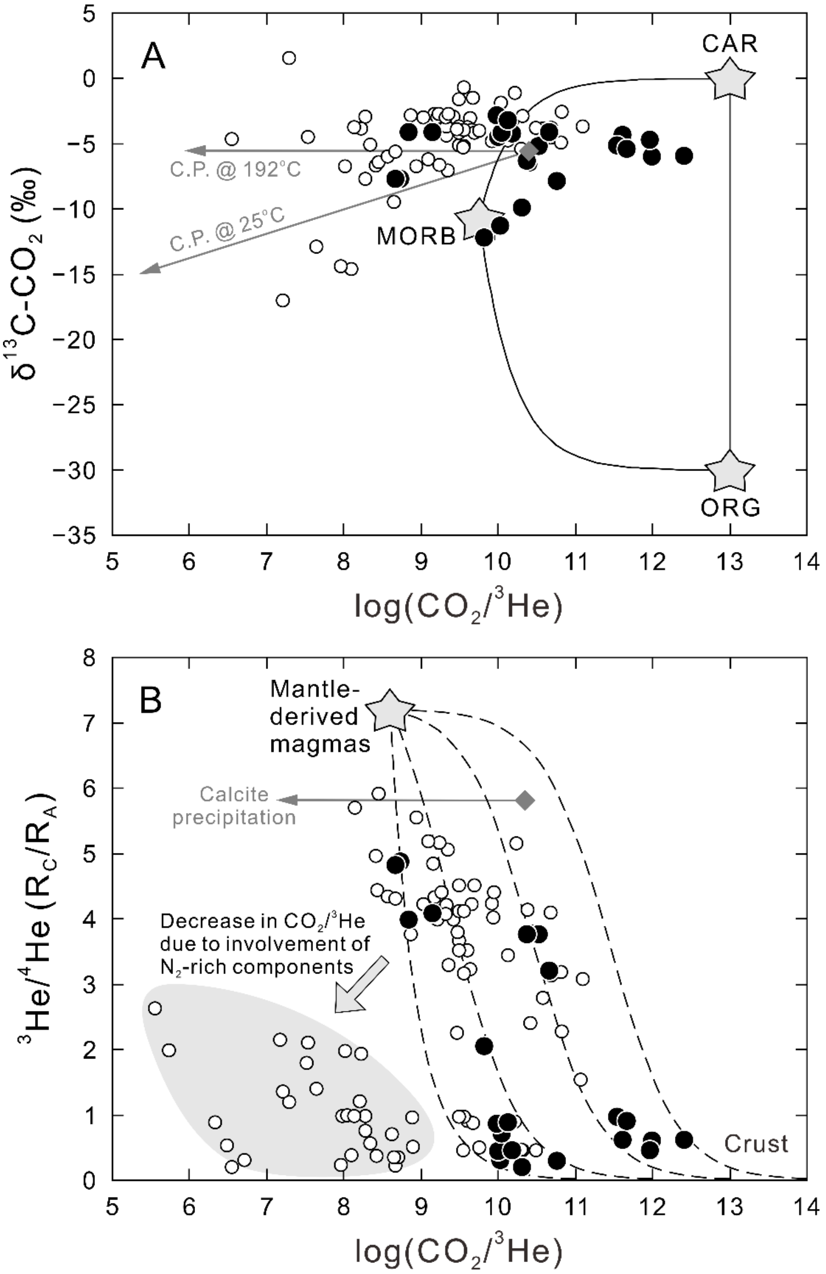

Supplementary Fig. 5. He-CO_2 systematics of fluids from the Tengchong volcanic field. MORB, depleted mantle; CAR, carbonate; ORG, organic matter; SED, sediments; C.P., calcite precipitation. (A) $\delta^{13}C$-CO_2 (‰) versus log($CO_2/^3He$). (B) $^3He/^4He$ (R_C/R_A) versus log($CO_2/^3He$). Reference values for parameters of the CAR and ORG end-members are given in the Supplementary Materials and Methods. Mixing methods are from Sano and Marty[7], and O'Nions & Oxburgh[18], and Javoy et al.[19]. Filled and open symbols represent samples in this study (Supplementary Data 1) and literature (Supplementary Data 2), respectively.

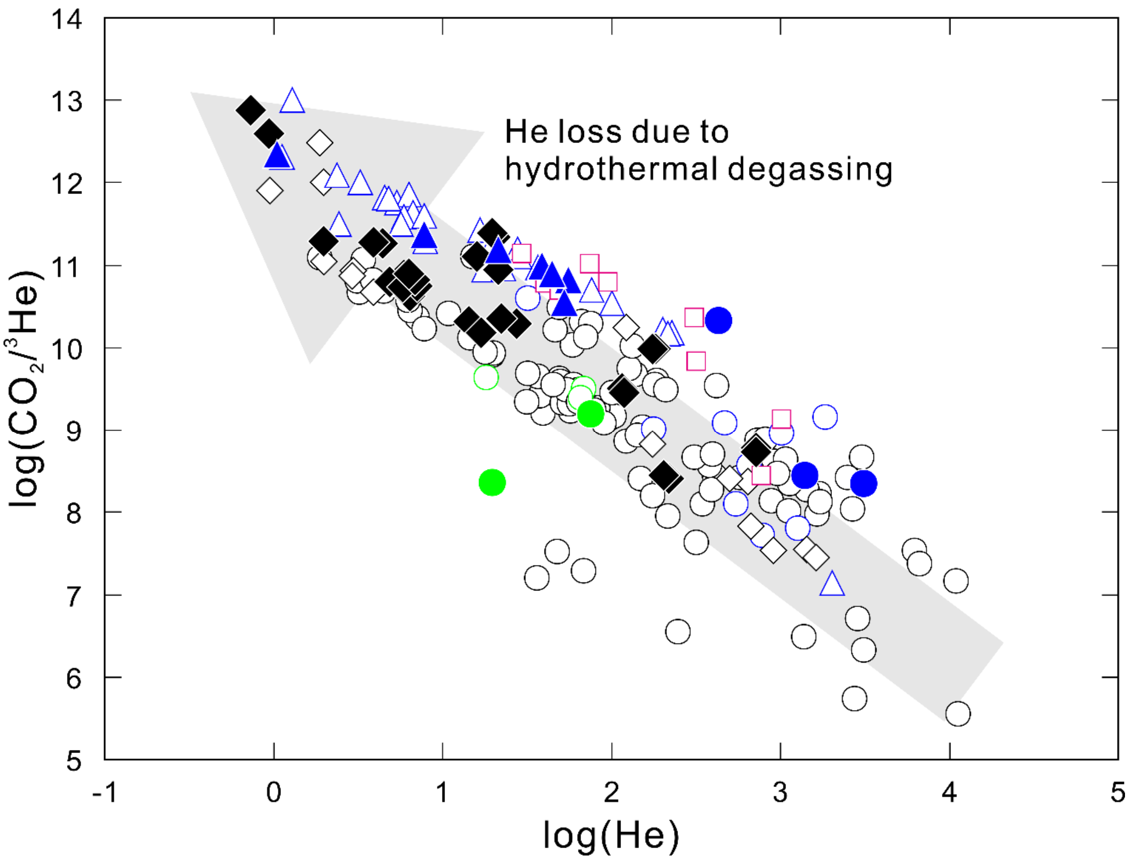

Supplementary Fig. 6. Plot of log(CO_2/^{3}He) versus log(He) showing possible influence of hydrothermal degassing on fractionation between He and CO_2. Figure symbols are as in Fig. 2 of the main text. Grey-shaded arrow denotes He loss due to hydrothermal degassing.

Supplementary References (1–19)

1. D. R. Hilton, T. P. Fischer, B. Marty, Noble gases and volatile recycling at subduction zones. Rev. Mineral. Geochem. 47, 319–370 (2002).
2. Y. Sano, T. P. Fischer, "The analysis and interpretation of noble gases in modern hydrothermal systems" in The Noble Gases as Geochemical Tracers, P. Burnard, Ed. (Advances in Isotope Geochemistry, Springer, Berlin Heidelberg, 2013), chap. 10, pp. 249-317.
3. Y. Yang, X. Zhou, Z. Guan, X, Rui. Hydrogeochemical characteristics of groundwater in seismic observation wells in the western Sichuan. Bulletin of Mineralogy, Petrology, Geochemistry 38, 966–976 (2019).
4. Weiss, R.F., 1971. Solubility of helium and neon in water and seawater. Journal of Chemical and Engineering Data 16, 235-241.
5. Graham, D.W., 2002. Noble gas isotope geochemistry of mid-ocean ridge and ocean island basalts: characterization of mantle source reservoirs. Reviews in Mineralogy and Geochemistry 47, 247-319.
6. Ballentine, C. J., Burgess, R. & Marty, B. Tracing fluid origin, transport and interaction in the crust. Rev. Mineral. Geochem. 47, 539–614 (2002).
7. Sano, Y. & Marty, B. Origin of carbon in fumarolic gas from island arcs. Chem. Geol. 119, 265–274 (1995).
8. Sano, Y., Takahata, N., Nishio, Y., Fischer, T.P. & Williams, S.N., Volcanic flux of nitrogen from the Earth. Chem. Geol. 171, 263–271 (2001).
9. Fischer, T.P. et al. Subduction and recycling of nitrogen along the central American margin. Science 297, 1154–1157 (2002).
10. Ellis, A.J., Golding, R.M., 1963. The solubility of carbon dioxide above 100 degrees C in water and in sodium chloride solutions. American Journal of Science 261, 47.
11. Ozima, M., Podosek, F.A., 2002. Noble gas geochemistry. Cambridge University Press.
12. Vogel, J.C., Grootes, P.M. & Mook, W.G., 1970. Isotopic fractionation between gaseous and dissolved carbon dioxide. Z. Physik 230, 225–238.
13. Mook, W.G., Bommerson, J.C., Staverman, W.H., 1974. Carbon isotope fractionation between dissolved bicarbonate and gaseous carbon dioxide. Earth and Planetary Science Letters 22, 169-176.
14. Kreemer, C., Blewitt, G. & Klein, E. C. A geodetic plate motion and global strain rate model. Geochem. Geophys. Geosyst. 15, 3849–3889 (2014).
15. Z. Huang, P. Wang, M. Xu, L. Wang, Z. Ding, Y. Wu, M. Xu, N. Mi, D. Yu, H. Li, Mantle structure and dynamics beneath SE Tibet revealed by new seismic images. Earth Planet. Sci. Lett. 411, 100–111 (2015).
16. C.-Y. Wang, H. Lou, P. G. Silver, L. Zhu, L. Chang, Crustal structure variation along 30°N in the eastern Tibetan Plateau and its tectonic implications. Earth Planet. Sci. Lett. 289, 367–376 (2010).
17. Wang, W., Wu, J., Fang, L., Lai, G. & Cai, Y. Crustal thickness and Poisson's ratio in southwest China based on data from dense seismic arrays. J. Geophys. Res. Solid Earth 122, 7219–7235 (2017).
18. R. K. O'Nions, E. R. Oxburgh, Helium, volatile fluxes and the development of continental crust. Earth Planet. Sci. Lett. 90, 331–347 (1988).
19. M. Javoy, F. Pineau, I. Iiyama, Experimental determination of the isotopic fractionation between gaseous CO_2 and carbon dissolved in tholeiitic magma. Contrib. Mineral. Petr. 67, 35–39 (1978).
