# Supplementary material for: Linking deeply-sourced volatile emissions to plateau growth dynamics in southeastern Tibetan Plateau
Source: Nat Commun. 2021 Jul 6;12:4157. doi: 10.1038/s41467-021-24415-y (PMC8260613; doi:10.1038/s41467-021-24415-y)
Supplement: Supplementary file 3 — Description of Additional Supplementary Files [file 41467_2021_24415_MOESM3_ESM.pdf]

Description of Additional Supplementary Files

File name: Supplementary Data 1
Description: He-C-N isotope and compositional data of fluids from the SETP and adjacent region (this study).

File name: Supplementary Data 2
Description: He-C isotope data of fluids from the SETP and adjacent region (literature).

File name: Supplementary Data 3
Description: Average 3He/4He, strain rates, and distances to the IACB for each sampling site of major active faults.

File name: Supplementary Data 4
Description: Initiation ages of major active faults in the study area.

File name: Supplementary Data 5
Description: Ages of late Cenozoic volcanic rocks in the study area.
